# Identification of the Biomarkers and Pathological Process of Heterotopic Ossification: Weighted Gene Co-Expression Network Analysis

**DOI:** 10.3389/fendo.2020.581768

**Published:** 2020-12-17

**Authors:** Shuang Wang, Jun Tian, Jianzhong Wang, Sizhu Liu, Lianwei Ke, Chaojiang Shang, Jichun Yang, Lin Wang

**Affiliations:** Shangnan County Hospital, Shangnan County, Shangluo City, China

**Keywords:** heterotopic ossification, biomarkers, WGCNA, pathological process, hub genes

## Abstract

Heterotopic ossification (HO) is the formation of abnormal mature lamellar bone in extra-skeletal sites, including soft tissues and joints, which result in high rates of disability. The understanding of the mechanism of HO is insufficient. The aim of this study was to explore biomarkers and pathological processes in HO+ samples. The gene expression profile GSE94683 was downloaded from the Gene Expression Omnibus database. Sixteen samples from nine HO- and seven HO+ subjects were analyzed. After data preprocessing, 3,529 genes were obtained for weighted gene co-expression network analysis. Highly correlated genes were divided into 13 modules. Finally, the cyan and purple modules were selected for further study. Gene ontology functional annotation and Kyoto Encyclopedia of Genes and Genomes pathway enrichment indicated that the cyan module was enriched in a variety of components, including protein binding, membrane, nucleoplasm, cytosol, poly(A) RNA binding, biosynthesis of antibiotics, carbon metabolism, endocytosis, citrate cycle, and metabolic pathways. In addition, the purple module was enriched in cytosol, mitochondrion, protein binding, structural constituent of ribosome, rRNA processing, oxidative phosphorylation, ribosome, and non-alcoholic fatty liver disease. Finally, 10 hub genes in the cyan module [actin related protein 3 (*ACTR3*), ADP ribosylation factor 4 (*ARF4*), progesterone receptor membrane component 1 (*PGRMC1*), ribosomal protein S23 (*RPS23*), mannose-6-phosphate receptor (*M6PR*), WD repeat domain 12 (*WDR12*), synaptosome associated protein 23 (*SNAP23*), actin related protein 2 (*ACTR2*), siah E3 ubiquitin protein ligase 1 (*SIAH1*), and glomulin (*GLMN*)] and 2 hub genes in the purple module [proteasome 20S subunit alpha 3 (*PSMA3*) and ribosomal protein S27 like (*RPS27L*)] were identified. Hub genes were validated through quantitative real-time polymerase chain reaction. In summary, 12 hub genes were identified in two modules that were associated with HO. These hub genes could provide new biomarkers, therapeutic ideas, and targets in HO.

## Introduction

Heterotopic ossification (HO) is the formation of abnormal mature lamellar bone in extra-skeletal sites, including soft tissues and joints (1). HO was first described 1,000 years ago in the healing of fractures (2). HO is a frequent complication associated with arthroplasties [occurs in up to 40% of cases (3)], traumatic brain and spinal cord injuries [occurs in up to 50% of cases (3, 4)], extensive burns, severe trauma and combat-related extremity injuries (5). Other research reveals that up to 75% of HO cases are associated with trauma (6).

To date, the only available prophylactic treatments for advanced HO are nonsteroidal anti-inflammatory drug (NSAID) treatments and localized low-dose irradiation. However, their action is not against precise molecular biological and genetic targets (7). At later stages, surgical excision is the only effective procedure for treatment, which includes many risks, such as wound healing complications, delayed therapy, rehabilitation, and recurrence (5). Therefore, early diagnosis, and new and effective therapeutic strategies urgently need to be developed.

Due to the development of genetic testing technology in recent years, an increasing number of studies focus on the inhibition of local factors and signaling pathways, such as bone morphogenetic protein (BMP) inhibitors, like noggin (8), BMP type 1 receptor inhibitor (8, 9), and nuclear retinoid acid receptor-gamma (RARγ) agonists (10). These inhibitors catalyze ectopic bone formation to provide earlier diagnoses and to develop treatments that are more effectives (11). Weighted gene co-expression network analysis (WGCNA), a new systems biology method, has been developed to analyze gene expression microarray profiling data (12). WGCNA describes how genes work interactively and provides insights into the correlation between heterogeneous traits and genetics on the basis of genetic networks (12). It has been widely used in identifying biomarkers and candidate genes in complex diseases (13). Thus, based on expression profiling of array data, WGCNA was applied to analyze the GSE94683 datasets in the Gene Expression Omnibus (GEO) database to identify specific HO-related genes, and provided an opportunity to improve the understanding of HO.

## Materials and Methods

### Data Collection and Preprocessing

The expression profiling of the array dataset GSE94683, which is a transcriptome analysis of HO mesenchymal stromal cells (MSCs), was shared on the GEO database (https://www.ncbi.nlm.nih.gov/geo/query/acc.cgi?acc=GSE94683). The research was performed on 16 individuals (9 HO- and 7 HO+) based on the GPL10630 platform. Transcriptome analysis was performed on mesenchymal stromal cells from HOs and amplified *in vitro* after two passages. Agilent Whole Human Genome Oligo Microarrays were used to compare expression profiles of MSCs from HO- bone marrow of healthy donors. Series matrix files and the platform file were downloaded (https://www.ncbi.nlm.nih.gov/geo/query/acc.cgi?acc=GSE94683). The series matrix file was preprocessed to identify differentially expressed genes (DEGs) using GEO2R online (https://www.ncbi.nlm.nih.gov/geo/geo2r/?acc=GSE94683) (14, 15). The probe ID was converted into a gene symbol through the platform file and the series matrix file.

### WGCNA on DEGs

The “WGCNA” package (12) in R software (version 4.0.0) was used for the network construction. Samples were corrected with limma package using the normalizeBetweenArrays function. A log2 transform was applied to the expression matrix of DEGs. Check missing values and filter (meanFPKM = 0.5) was conducted to evaluate the expression matrix file. Finally, a total of 3529 genes were obtained for subsequent analysis. WGCNA was used to cluster the DEGs into differently colored modules using the dissimilarity measure (1-topological overlap measure (TOM), TOM ≥ 0.15) (16). If the eigenvalue correlation coefficients of different modules were greater than 0.25, the different modules were integrated into one module (17). Module−trait relationships (*p* < 0.05) were used to determine HO-related modules. GeneTraitSignificance (GS) and geneModuleMembership (MM) were determined after relating modules to external clinical traits. All merged modules were exported for further research.

### Gene Ontology (GO) and Pathway Enrichment Analysis of HO-Related Modules

HO-related modules were analyzed online using the database for annotation, visualization and integrated discovery (https://david.ncifcrf.gov/summary.jsp) (18) for GO functional annotation and Kyoto Encyclopedia of Genes and Genomes (KEGG) pathway enrichment analysis; *p* < 0.05 indicated significant differences. Then, GO was visualized using ggplot2 and the Cairo package in the R software, while KEGG was visualized using stringr and the Cairo package in the R software.

### Protein–Protein Interaction (PPI) Network Construction

The online search tool for the retrieval of interacting genes (STRING; version 11.0; www.string-db.org) provides information for known and predicted PPIs (19). In this study, STRING was used to analyze the PPIs among HO-related modules. All genes in the HO-related modules were analyzed using the STRING online website. A confidence score >0.7 has been chosen to construct the PPI network in Cytoscape software (Version 3.7.2) (20). Genes with a node degree >10 are considered to be the key genes in the PPI network (15).

### Identification of Hub Genes

The key genes in HO-related modules have been determined through an absolute value of the MM >0.8 and GS >0.2 (15). PPI network key genes were determined using a node degree >10. The genes that were common components between key genes in HO-related modules and key genes in the PPI network were considered to be hub genes and were visualized using Venn diagrams (results were reconstructed by Excel 2016) (21).

### HO and Normal Sample Collection, Separating MSCs, Extraction of Total RNA, and Quantitative Real-time Polymerase Chain Reaction (qRT-PCR)

Four HO+ samples came from four HO patients (8 sample characteristic show in **Table 1**) surgically treated at the Shangnan County Hospital, Shangnan County, Shaanxi Province, China. Four normal (HO-) samples came from the bone marrow of four healthy donors at Shangnan County Hospital. After tissue harvested, the tissues of HO ample were separated into 1 mm3 and digested by collagenase 2. MSCs were separated by gradient centrifugation through Percoll (22, 23) (density 1.073 g/ml, Solarbio, China). MSCs were cultured in dulbecco’s modified eagle medium containing 10% fetal bovine serum (Bioind, Kibbutz Beit Haemek, Israel), 1% nonessential amino acid (Hyclone, Logan, UT, USA), 1% antibiotics (Sigma, St. Louis, MO, USA), 10 ng/ml epidermal growth factor (Gibco, Carslbad, CA, USA), 10 ng/ml fibroblast growth factor (Gibco, Carslbad, CA, USA) (24), and were trypsinized once confluent and propagated to 2 passage. After harvested MSCs were used to extract total RNA using the TRIzol™ reagent (Invitrogen, USA), according to the manufacturer’s protocol. Total RNA of all samples was stored in liquid nitrogen after extraction. For each sample, 1 μg of RNA was converted to cDNA using a First Strand cDNA Synthesis Kit (Takara Bio, Dalian, China). All primers (12 hub genes and β-actin) are shown in **Supplementary Table 1**, and have been designed and validated by the Takara Biological Company. Template cDNA (10 ng/rxn), SYBR Green fluorescent reagent (Tli RNaseH Plus, Takara Bio, Dalian, China) and a Roche LightCycler^®^ 480 II (Roche, Switzerland) were utilized for qRT-PCR analysis. The reaction conditions were as follows: an initial denaturation at 95°C for 5 min, denaturation at 95°C for 30 s, annealing at 58°C for 30 s, extension at 72°C for 30 s, for a total of 45 cycles. Relative gene expression was calculated using the 2 *^−△△CT^* method with *β*-actin (*ACTB*) as the endogenous control gene. This study was approved by the ethics committee of Shangnan County Hospital. The study was conducted in accordance with the Declaration of Helsinki and all patients provided written informed consent before the study. All data were de-identified, presented as the mean ± standard deviation (SD), and analyzed using GraphPad Prism 8.01 software (GraphPad Software, USA). Statistical significance was determined using the Student’s *t*-test and *p <*0.05 was considered a significant difference.

**Table 1:** The detail clinical information of eight samples.

Sample	Gender	Age	BMI (kg/m2)	Sample location
HO+1	male	63	22.3	intertrochanter of left femur (8 M after fracture)
HO+2	male	61	21.2	intertrochanter of left femur (6 M after fracture)
HO+3	female	65	19.8	intertrochanter of right femur (7 M after fracture)
HO+4	female	60	23.6	intertrochanter of right femur (10 M after fracture)
HO-1	female	62	22.4	right anterior superior iliac spine bone marrow biopsy
HO-2	male	62	21.2	right anterior superior iliac spine bone marrow biopsy
HO-3	male	65	23.6	right anterior superior iliac spine bone marrow biopsy
HO-4	female	61	19.4	right anterior superior iliac spine bone marrow biopsy

## Results

### Processing of DEGs and Co-Expression Network Construction

**Figure 1:**
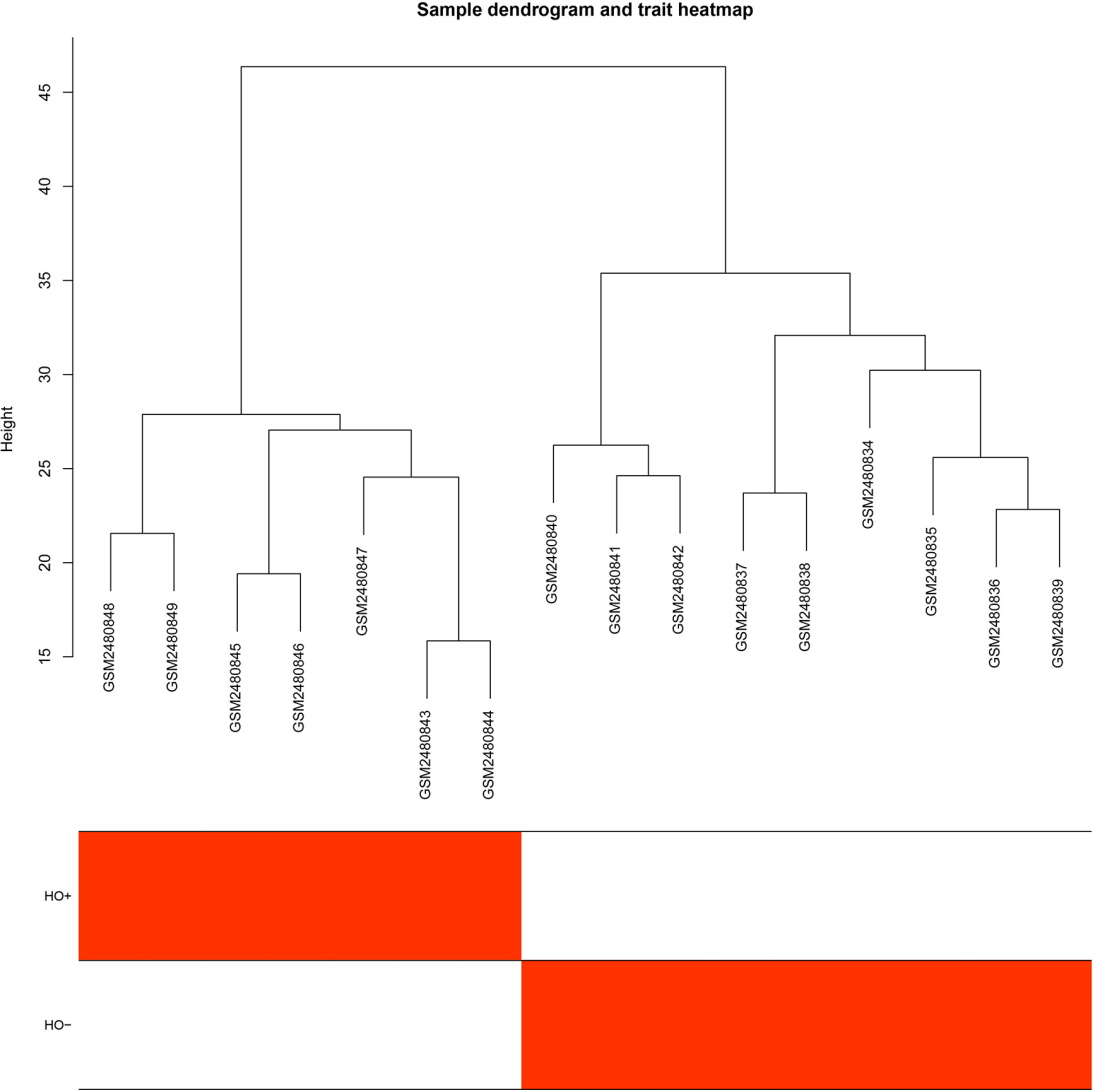
Sample dendrogram and trait heatmap.

**Figure 2:**
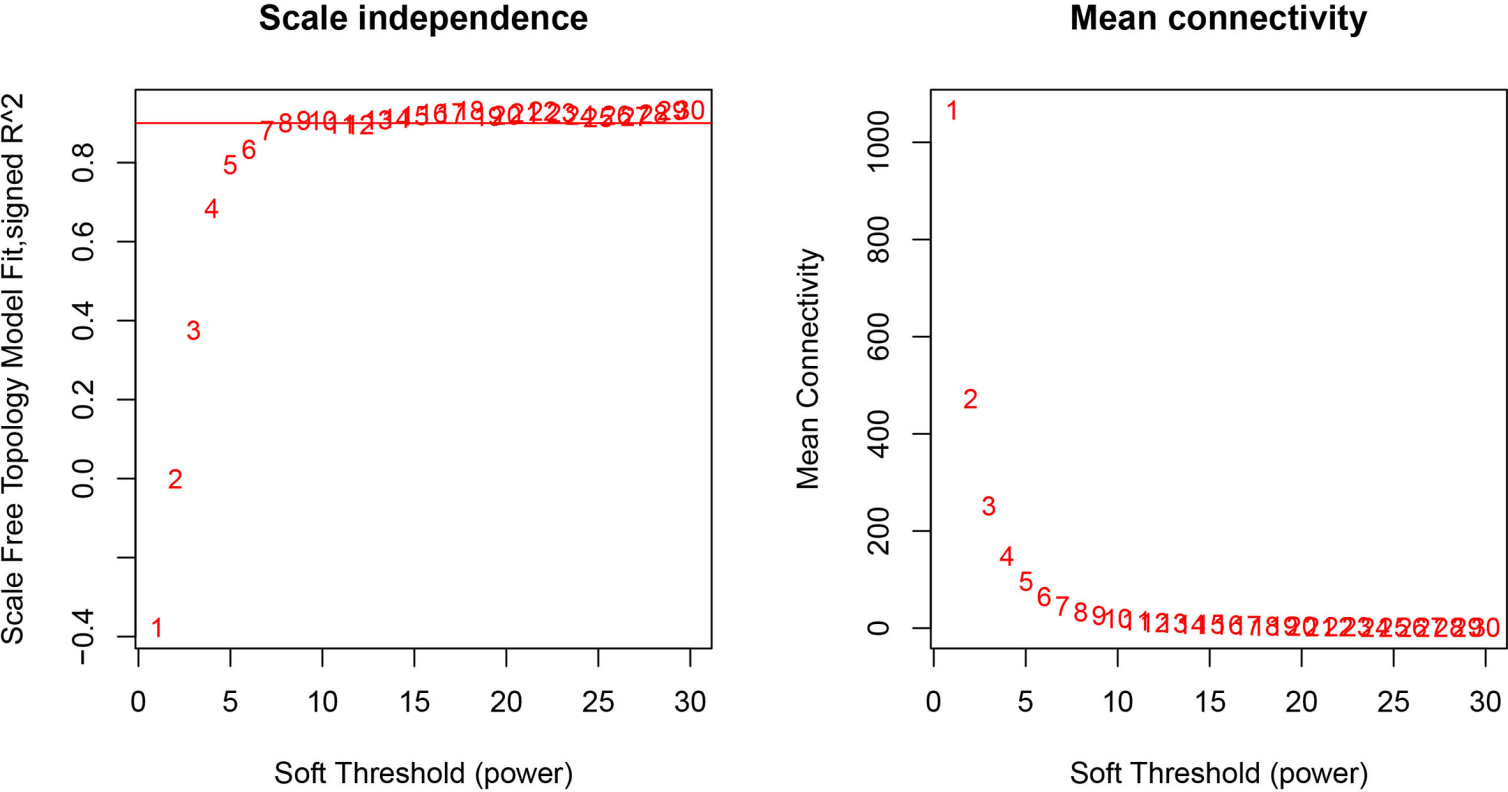
Analysis of network topology for various soft-thresholding powers. The left panel shows the scale-free fit index, signed Rˆ 2 (y-axis) and the soft threshold power (x-axis). *β* = 9 has been chosen for subsequent analysis. The right panel shows the mean connectivity (y-axis), which is a strictly decreasing function of the power *β* (x-axis).

**Figure 3:**
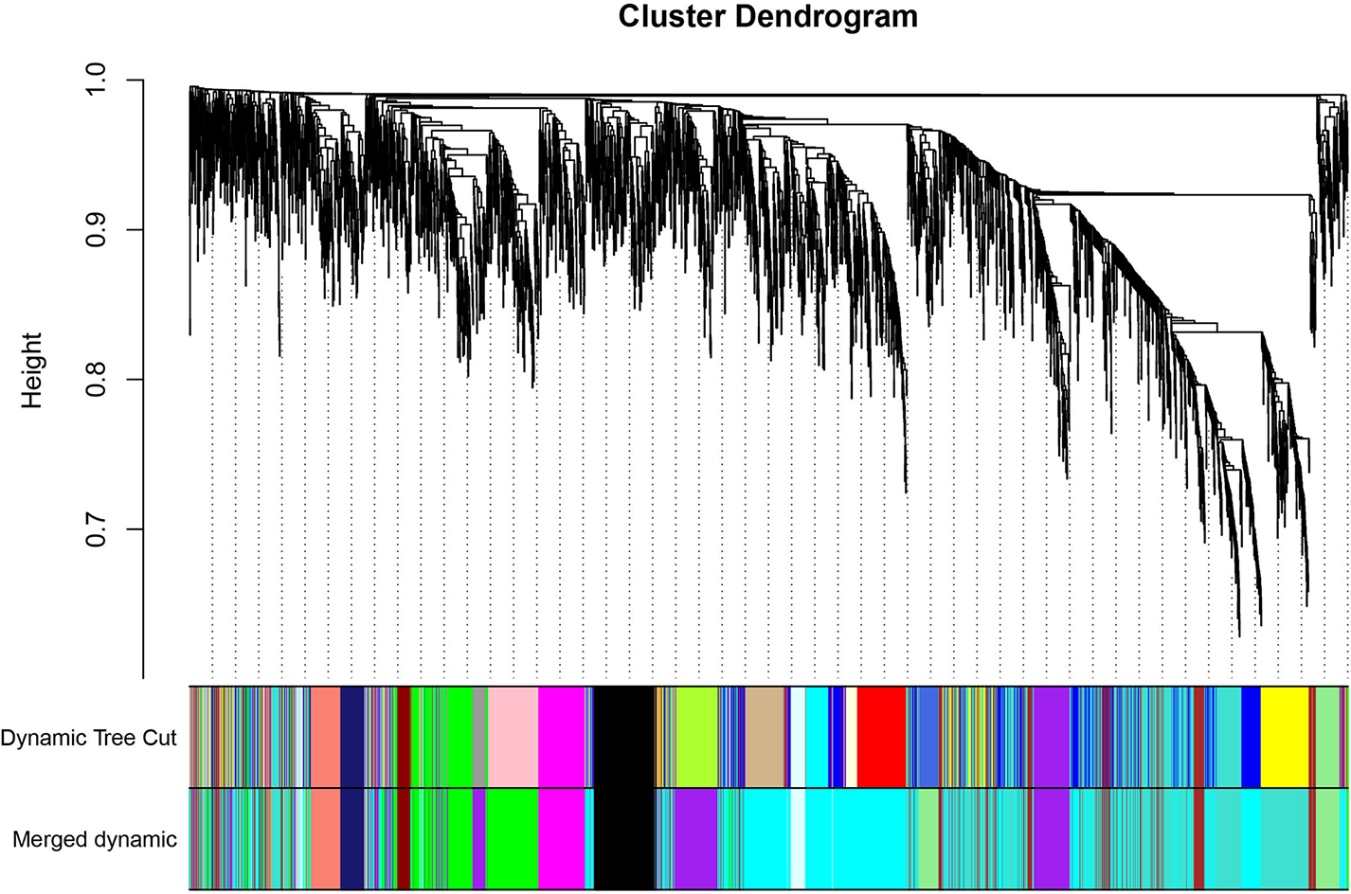
Clustering dendrogram of genes. The color bands provide a simple visual comparison of module (and merged dynamic) assignments (branch cuttings) based on the dynamic tree cutting method.

The GEO2R online analysis tool was used to detect DEGs between HO+ and HO- samples, and then the adjusted *p*-value (adjusted using the Benjamini & Hochberg method) was calculated. Genes that met the cutoff criteria, adjusted *p <*0.05, were considered to be HO-related DEGs. Through check missing value and filter, a total of 3,529 genes were selected as HO-related DEGs. All HO-related DEGs were analyzed in R software through the WGCNA package. There are no sample outliers depicted in **Figure 1**. In this study, the soft-thresholding parameter has been determined as *β* = 9, where the curve first reached Rˆ2 = 0.9, to construct a weighted network based on a scale-free topology criterion (**Figure 2**) (15). As shown in **Figure 3**, 22 modules are detected by the dynamic tree cutting method, then 22 modules are merged into 13 new modules through eigenvalue correlation coefficients of different modules greater than 0.25 (17).

### Identification of HO-Related Modules and Functional Annotation

**Figure 4:**
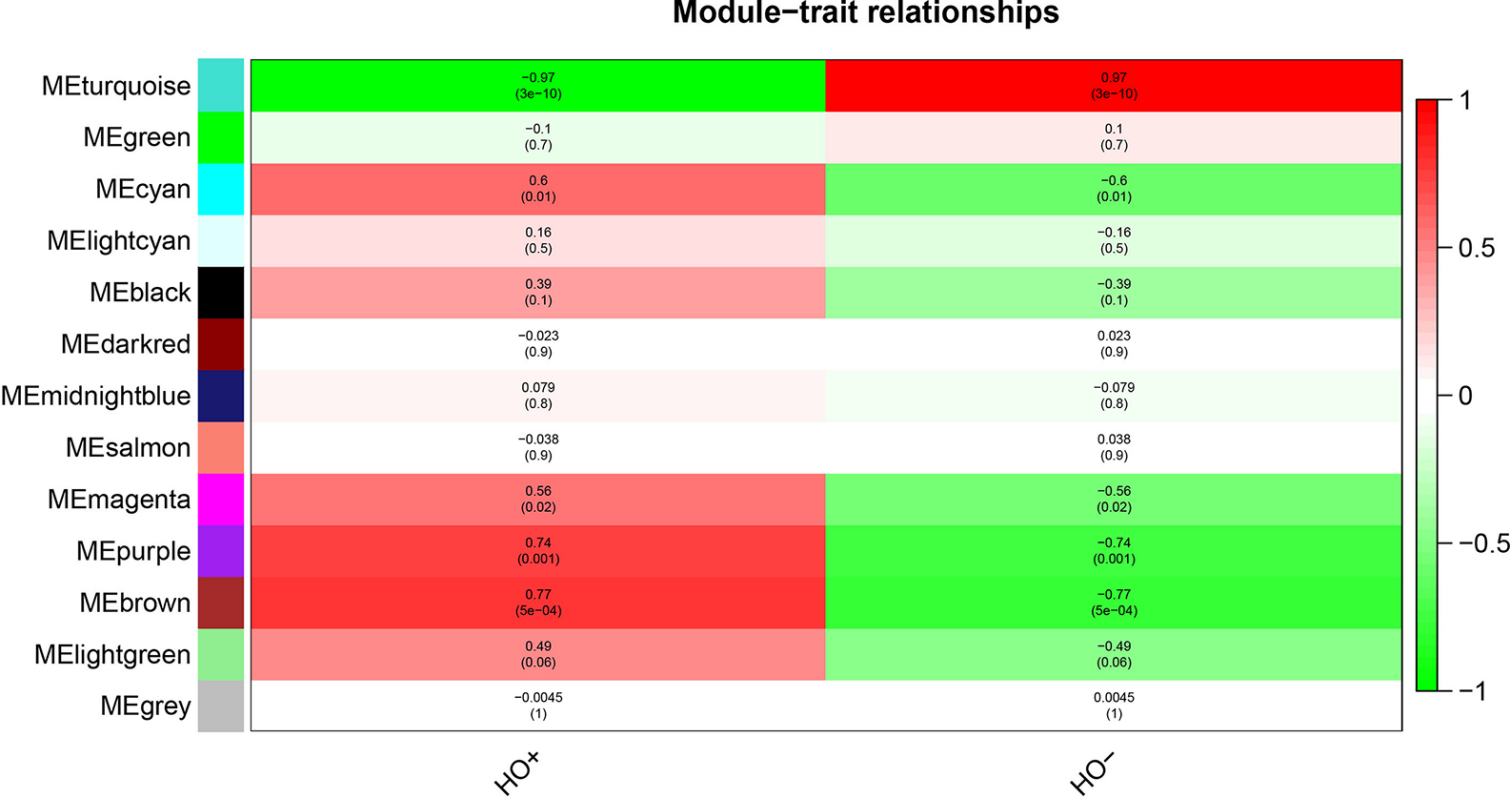
Module-trait relationships show that cyan, magenta, purple, and brown modules are related to HO.

**Figure 5:**
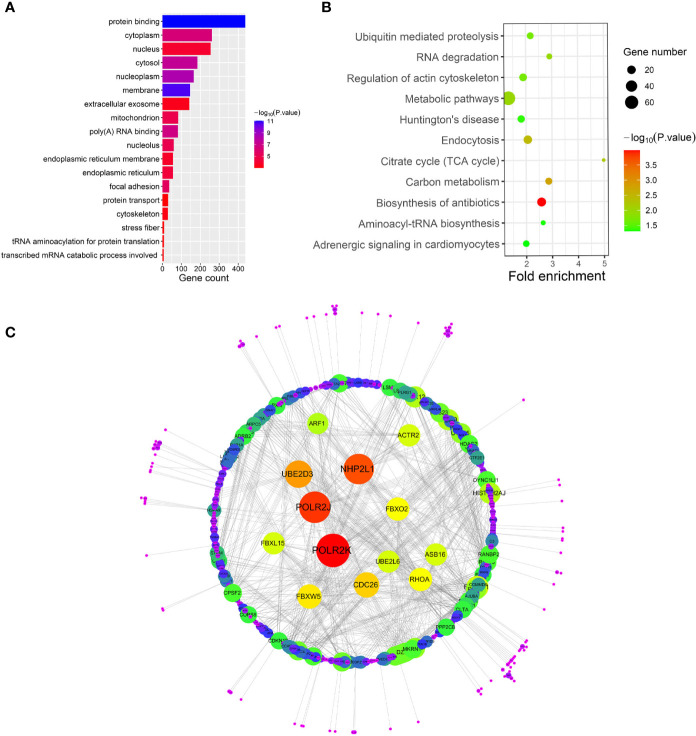
**(A–C)** show gene ontology (GO), Kyoto Encyclopedia of Genes and Genomes (KEGG) pathway and protein-protein interaction (PPI) analyses of the cyan module, respectively.

**Figure 6:**
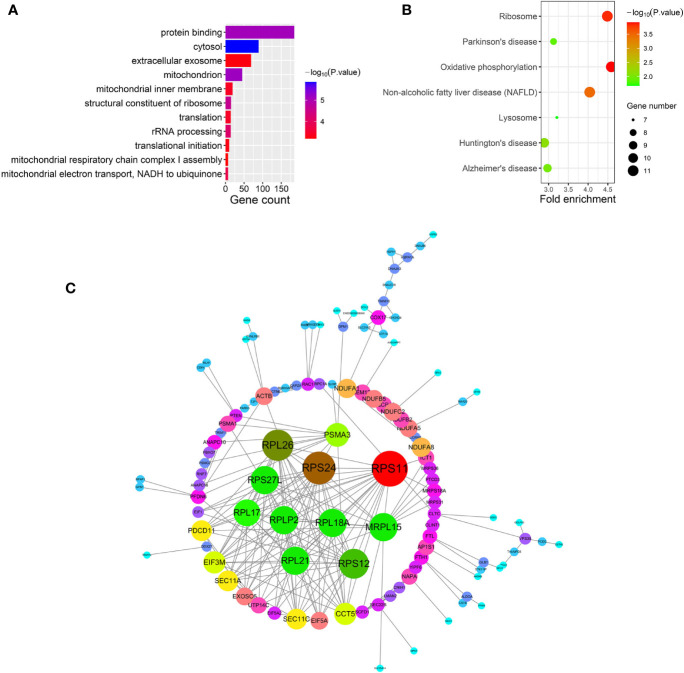
**(A–C)** show gene ontology (GO), Kyoto Encyclopedia of Genes and Genomes (KEGG) pathway and protein-protein interaction (PPI) analyses of the purple module, respectively.

After relating modules to traits, high correlations were observed in HO traits (**Figure 4**). Four modules (cyan *r* = 0.6, *p* = 0.01; magenta *r* = 0.56, *p* = 0.02; purple *r* = 0.74, *p* = 0.001; and brown *r* = 0.77, *p* = 5e−04) were selected as HO-related modules through the *p* < 0.05 criterion. Because the brown and magenta modules did not have a meaningful functional annotation analysis result, the brown and magenta modules were removed for subsequent analysis. **Figure 5A** shows that the cyan module is enriched in a variety of components, including protein binding, membrane, nucleoplasm, cytosol, and poly(A) RNA binding, based on GO analysis (*p* < 0.001). **Figure 5B** shows that the cyan module includes genes involved in the biosynthesis of antibiotics, carbon metabolism, endocytosis, citrate cycle (TCA cycle), and metabolic pathways, based on KEGG pathway analysis (*p* < 0.05). **Figure 6A** shows that the purple module is enriched in a variety of components, including cytosol, mitochondrion, protein binding, structural constituent of the ribosome, and rRNA processing, based on GO analysis (*p* < 0.001). **Figure 6B** shows that the purple module includes genes involved in oxidative phosphorylation, ribosome, non-alcoholic fatty liver disease (NAFLD), Huntington’s disease, and Alzheimer’s disease, based on KEGG pathway analysis (*p* < 0.05). Finally, the cyan module, with 887 genes, and the purple module, with 365 genes, were deemed to be clinically significant modules with HO associations.

### PPI Network Analysis

To investigate the interactive relationships among DEGs, we submitted the DEGs to the STRING database with a combined score >0.7. Cytoscape software has been used to construct PPI networks (20). Nodes with a higher degree of connectivity tend to be more essential in the functional network (15). The genes with a node degree >10 were identified as key genes in the PPI analysis. The PPI network in the cyan module is shown in **Figure 5C**; there are 76 key genes in the cyan module by PPI analysis, as shown in **Supplementary Table 2**. The PPI network in the purple module is shown in **Figure 6C**; there are 13 key genes in the purple module using PPI analysis, as shown in **Supplementary Table 3**.

### Identification of Hub Genes

**Figure 7:**
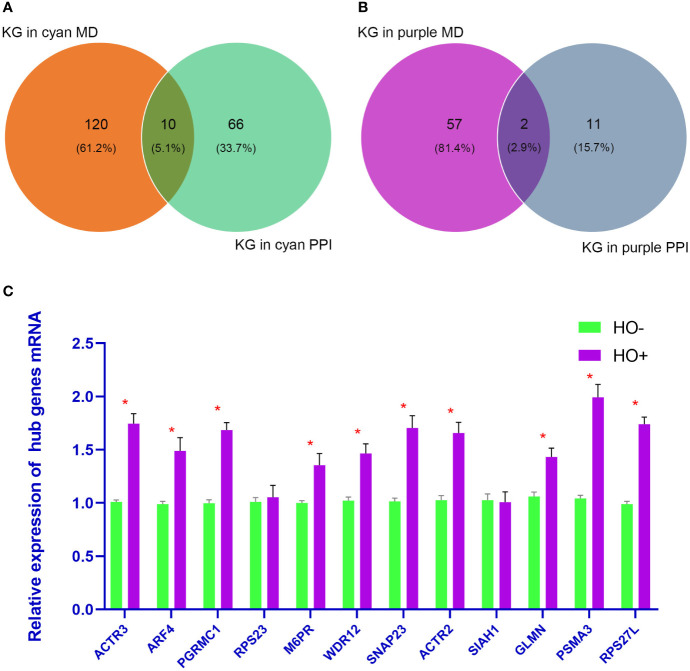
**(A, B)** show the Venn diagram analysis of the cyan and purple modules, respectively. **(C)** shows the quantitative real-time polymerase chain reaction analysis of 12 hub genes. *p <*0.05 is considered statistically significant. KG, Key genes; MD, modules.

Based on the absolute value of the MM >0.8 and GS >0.2, 130 key genes and 59 key genes were filtered from the cyan and purple modules, respectively (**Supplementary Tables 4** and **5**). The final hub genes are considered to be common genes between key genes in the cyan and purple modules and key genes in the cyan and purple PPI analysis (15). Through Venn diagram analysis, there were 10 hub genes [actin related protein 3 (*ACTR3*), ADP ribosylation factor 4 (*ARF4*), progesterone receptor membrane component 1 (*PGRMC1*), ribosomal protein S23 (*RPS23*), mannose-6-phosphate receptor (*M6PR*), WD repeat domain 12 (*WDR12*), synaptosome associated protein 23 (*SNAP23*), actin related protein 2 (*ACTR2*), siah E3 ubiquitin protein ligase 1 (*SIAH1*), and glomulin (*GLMN*)] in the cyan module (**Figure 7A**) and 2 hub genes in the purple module [proteasome 20S subunit alpha 3 (*PSMA3*) and ribosomal protein S27 like (*RPS27L*)] (**Figure 7B**).

### Validation of Hub Genes through qRT-PCR

Four HO tissue samples and four normal samples were used to evaluate the expression levels of the 12 hub genes by qRT-PCR analysis. As shown in **Figure 7C**, mRNA levels of *ACTR3*, *ARF4*, *PGRMC1*, *M6PR*, *WDR12*, *SNAP23*, *ACTR2*, *GLMN*, *PSMA3*, and *RPS27L* were significantly upregulated in HO than in normal samples (*p* < 0.05). However, the other two genes (*RPS23* and *SIAH1*) showed no significant difference.

## Discussion

Because of the high incidence [up to 90% after certain types of hip arthroplasty or acetabular fractures (3, 25, 26)], low quality of life, and the resulting disability caused by HO, many studies have tried to identify causal factors from the mechanism of injury, management of wounds and complications, and the subsequent formation of HO (2, 27). The detailed molecular biological mechanism is not fully understood. The treatment of HO is restricted to the use of NSAIDs, radiation, and surgical excision (2). Although NSAIDs have demonstrated prophylactic efficacy against HO, it is confirmed that HO prophylaxis with indomethacin increases the risk of long bone nonunion (28, 29). The existing literature shows that rates of HO after hip arthroplasty decrease to 25% after radiation therapy compared with a range of approximately 5 to 90% before treatment (30, 31). However, radiation therapy has not been adequately studied at other joints than the hip (32). Additional side effects have been reported in a prospective, randomized study, including progressive soft-tissue contracture, delayed wound-healing, nonunion, inhibited ingrowth of press-fit hip implants, risk of malignancy, and others (32, 33). Surgical excision is also effective therapy for the treatment of previously existing HO, but has risks of delayed wound healing, infection, nerve injury, and recurrent contracture (34, 35). The investigation of pathological processes, clinical manifestation, diagnosis, treatment, and prevention is particularly essential and urgent.

In this study, HO-related biomarkers and pathological processes were explored using the WGCNA algorithm in R software. Through WGCNA analysis, it was found that both the cyan and purple modules were closely related to HO. Specifically, the cyan module containing 887 genes played a key role in protein binding, membrane, nucleoplasm, cytosol, and poly(A) RNA binding. The cyan module contained components enriched in biosynthesis of antibiotics, carbon metabolism, endocytosis, citrate cycle (TCA cycle), metabolic pathways, and signaling pathways. Ten hub genes (*ACTR3*, *ARF4*, *PGRMC1*, *RPS23*, *M6PR*, *WDR12*, *SNAP23*, *ACTR2*, *SIAH1*, and *GLMN*) were selected by combining the PPI network analysis with that of genes identified in the cyan module.

A previous study showed that the TCA cycle and metabolic pathways are significantly changed in mice HO modules (36). However, this has not been reported in humans. Another recent bioinformatics study showed that HO-related DEGs are mainly associated with metabolic processes in a mouse burn/tenotomy-induced HO model (37), but this has not been reported in humans. *ACTR2* and *ACTR3* are components of the seven-subunit Arp2/3 complex, which plays a key role in generating branched actin filament networks during many different cellular processes (38). In recent studies, *ACTR3* has been confirmed to play key roles in the early immunodiagnosis of lung cancer and cholangiocarcinoma (39, 40); there is no evidence that *ACTR2* and *ACTR3* are related to HO. *ARF4* is a component of osteogenic transcription factors, and is a necessary transcription factor for gene expression of bone matrix proteins (osteoblast markers), such as osteocalcin (41, 42). *ARF4* plays key roles in the first and final stages of osteoblastic differentiation (42). Osteoblast differentiation is a key biological process for the occurrence and development of HO. Other research showed that ARF proteins control tumor proliferation and metastasis (43). Whether ARF4 protein plays a role in the proliferation of heterotopic ossification cells remains to be verified. Therefore, *ARF4* may play key roles in HO development. *PGRMC1*/Sigma-2 receptor is confirmed to play key roles in the function of tumor proliferation (such as breast tumors, lung adenocarcinoma cells, and ovarian cancer) and chemoresistance (44–47). Recent research showed that *PGRMC1* significantly suppresses the hydrogen peroxide-induced oxidative stress response in full-thickness fetal membrane explants and chorion cells (48). Another study showed that oxidative stress plays an important role in cancer bone demineralization, aortic valve mineralization, and HO in disease development (49). Thus, *PGRMC1* may work in HO by regulating the oxidative stress response. *RPS23* is involved in the protein synthesis processes and progression of disc degeneration (DD) (50), suggesting its potential use in the diagnosis and therapy of DD. Another study showed that *RPS23* is a hub gene in gastric cancer (51). There is no evidence that *RPS23* is related to HO. Similar to *RPS23*, it was found that *M6PR*, *WDR12*, *SIAH1*, and *GLMN* did not have a relationship with HO in previous studies. A previous study showed that *SNAP23* expression is detected in osteoblastic cells (52). However, there was no evidence showing that *SNAP23* influences the HO process.

The purple module had 365 genes that played key roles in cytosol, mitochondrion, protein binding, structural constituent of ribosome, and rRNA processing. The purple module also contained components enriched in oxidative phosphorylation, ribosome, NAFLD, lysosome, and signaling pathways. Two hub genes (*PSMA3* and *RPS27L*) were selected by combining the PPI network analysis with genes identified in the purple module. A recent bioinformatics study showed that HO-related DEGs are mainly associated with oxidative phosphorylation in a mouse burn/tenotomy-induced HO model (34), but in humans, this has not been confirmed. An interesting study showed that *RPS27L* is significantly upregulated after irradiation in human peripheral blood (53). Another study showed that *RPS27L* is a physiological regulator of p53 that suppresses genomic instability and tumorigenesis (54). Radiation therapy is an effective method in the treatment of HO, possibly regulated by *RPS27L*. We speculate that regulating *RPS27L* expression may achieve the same effect as radiation therapy. Previous research has shown overexpression of RPS27L within the physiological inducible levels promoted, whereas siRNA silencing of RPS27L inhibited, apoptosis induced by etoposide (55). Another research has shown reduced apoptosis of osteoprogenitors may be responsible for increased osteogenesis in severely-injured patients (56). In a previous study, *PSMA3* is not related to HO.

In this study, the qRT-PCR analysis showed that 10 hub genes (*ACTR3*, *ARF4*, *PGRMC1*, *M6PR*, *WDR12*, *SNAP23*, *ACTR2*, *GLMN*, *PSMA3*, and *RPS27L*) had significance between HO and normal groups, and further verifies the reliability of the WGCNA analysis.

It is worth noting that in this study, 8 (*ACTR3*, *ARF4*, *PGRMC1*, *RPS23*, *WDR12*, *ACTR2*, *SIAH1*, and *RPS27L*) of 12 hub genes are related to cancers, such as lung cancer, epithelial ovarian cancer, gastric cancer, glioblastoma, breast cancer, and esophageal cancer (51, 54, 57–63). A last year’s research showed pulmonary adenocarcinoma cells around the ossification expressed bone morphogenetic protein-2 and osteopontin, which generally induce and stimulate bone formation (64, 65). Another research showed overexpression of BMP-2 promote HO form in rectal cancer (66). Another research showed histogenesis of HO in this case of metastatic colon cancer is from the stromal cells in the tumoral microenvironment (67). HO occurs in a variety of tumors, thus, HO may have a similar molecular biology process as cancer.

**Figure 8:**
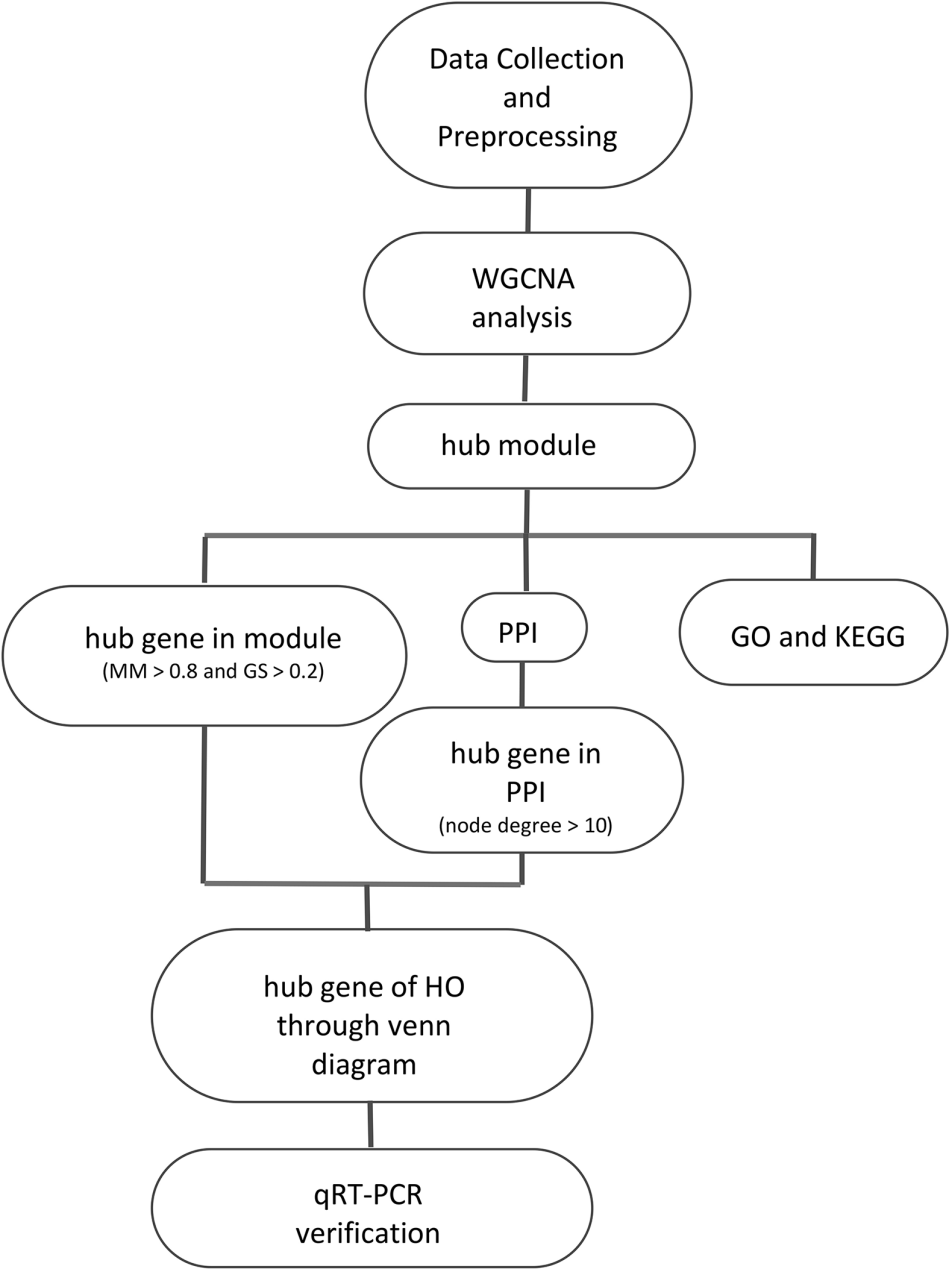
The workﬂow of the study.

There are several highlights of this study. First, this study is the first where MSCs were used for bioinformatics analysis of HO. Studying MSCs could contribute to a comprehensive understanding of HO. Second, WGCNA, which is used for the first time in HO analysis, has an advantage in processing gene expression datasets. This study not only confirmed the findings of previous studies, but also discovered new biomarkers for the further study of HO. However, the current study also has limitations. The sample size is small because there is no suitable data for analysis in the existing database, animal experimental verification has not been performed, and there are no additional clinical data (such as gender and age) in the original data. Future studies will further investigate HO in animal models. At last, we provide workﬂow showed in **Figure 8**.

## Conclusions

In this study, the WGCNA algorithm was used to process gene expression datasets and identified a cyan module with 10 hub genes and a purple module with 2 hub genes associated with HO. Targeting these hub genes could improve the understanding of the pathological processes of HO and provide new therapeutic ideas and targets.

## Data Availability Statement

The datasets presented in this article are available at https://www.ncbi.nlm.nih.gov/geo/query/acc.cgi?acc=GSE94683

## Ethics Statement

The studies involving human participants were reviewed and approved by Ethics Committee of Shangnan County Hospital and the ethics committee Shangnan County Hospital is affiliated to Shangnan County Hospital. The patients/participants provided their written informed consent to participate in this study. Written informed consent was obtained from the individual(s) for the publication of any potentially identifiable images or data included in this article.

## Author Contributions

JT, JW, SL, and LK conceived and designed the study. SW collected the data and wrote the manuscript. CS and JY performed the data analysis. LW and SW contributed to the language editing. All authors contributed to the article and approved the submitted version.

## Conflict of Interest

The authors declare that the research was conducted in the absence of any commercial or financial relationships that could be construed as a potential conflict of interest.
