# Supplementary material for: Identification of the Biomarkers and Pathological Process of Heterotopic Ossification: Weighted Gene Co-Expression Network Analysis
Source: Front Endocrinol (Lausanne). 2020 Dec 17;11:581768. doi: 10.3389/fendo.2020.581768 (PMC7774600; doi:10.3389/fendo.2020.581768)
Supplement: Supplementary file 1 [file Table_1.docx]

***Supplementary Material***

# Supplementary Table 1

| Genes | Primers |
| --- | --- |
| ACTR3 | F: TGGAAAGGTTTATGGAGCAAGTGAT  R: AAGCCTGGAACATTGAAGGACTC |
| ARF4 | F: ATAGTCACCACCATTCCTACCATTG  R: CTAATTCTATCTTGACCACCAACATCC |
| PGRMC1 | F: GAAGCACTGAAGGATGAGTACGATG  R: CACGTGATGATACTTGAAAGTGAACTGAG |
| RPS23 | F: GCAAGTGTCGTGGACTTCGTACTG  R: TGGCTGTTTGGCTTCAACTCCTACT |
| M6PR | F: TGGGTTCCATCTTACTTGTCACG  R: TGCTCCATTCCTTTGGCTCC |
| WDR12 | F: GAATGGAGCAGTTTCCCCACTTAGC  R: CACATTTCGAGGTTTAGAACGGCAG |
| SNAP23 | F: GGAGCAGCCAGTGGTGGATACAT  R: GTGTCAGCCTTGTCTGTGATTCGT |
| ACTR2 | F: CAGGCAGTTCTGACTTTGTACGCT  R: TATCCCTCCCAGCAATATCCAGTCT |
| SIAH1 | F: CCACACACAGAAAAAGCAGACCA  R: CATCATCACCCAGTCAACAGCAC |
| GLMN | F: GCTTGAAATGCCCTTTGCTGAC  R: GATATGCCAGAGAAGCCATTGAGTC |
| PSMA3 | F: GCATGTTGGAATGGCAGTAGCAG  R: GCAAGATGTTTTAGTGGAATGTTGTAGC |
| RPS27L | F: AAAAGAAACGCCTAGTACAAAGTCC  R: AAATGAACACCCTTCTGTGAGTCTG |
| *β*-actin | F: GTCCACACAGGGGAGGTGAT  R: GGGAGACCAAAAGCCTTCATACAT |

# Supplementary Table 2

**Supplementary Table 2** show key genes in PPI network in cyan module.

(Nodes > 10)

| symbol | NeighborhoodConnectivity | NumberOfDirectedEdges | Degree |
| --- | --- | --- | --- |
| POLR2K | 11.35484 | 31 | 31 |
| POLR2J | 11.86207 | 29 | 29 |
| NHP2L1 | 13.92857 | 28 | 28 |
| UBE2D3 | 14.04 | 25 | 25 |
| CDC26 | 16.21739 | 23 | 23 |
| FBXW5 | 16.72727 | 22 | 22 |
| RHOA | 6.428571 | 21 | 21 |
| FBXO2 | 16.71429 | 21 | 21 |
| ACTR2 | 10.75 | 20 | 20 |
| UBE2L6 | 16.1 | 20 | 20 |
| FBXL15 | 17.45 | 20 | 20 |
| FBXO31 | 17.45 | 20 | 20 |
| ASB16 | 17.45 | 20 | 20 |
| RPS20 | 11.52632 | 19 | 19 |
| ARF1 | 9.894737 | 19 | 19 |
| WDR12 | 10.21053 | 19 | 19 |
| HIST1H2AJ | 9 | 19 | 19 |
| UTP15 | 12.05556 | 18 | 18 |
| RNF4 | 17.5 | 18 | 18 |
| GLMN | 18.16667 | 18 | 18 |
| RPS23 | 11.64706 | 17 | 17 |
| SNAP23 | 8.882353 | 17 | 17 |
| DDX47 | 12.76471 | 17 | 17 |
| UBE4A | 18.23529 | 17 | 17 |
| SIAH1 | 18.52941 | 17 | 17 |
| RCHY1 | 18.35294 | 17 | 17 |
| DZIP3 | 18.29412 | 17 | 17 |
| UBE2H | 18.11765 | 17 | 17 |
| RPL36 | 10 | 16 | 16 |
| CDK7 | 11 | 16 | 16 |
| HDAC2 | 6.4375 | 16 | 16 |
| NEDD4 | 19.25 | 16 | 16 |
| ELAVL1 | 13.8125 | 16 | 16 |
| SRSF7 | 14.3125 | 16 | 16 |
| DYNC1LI1 | 10.3125 | 16 | 16 |
| RANBP2 | 8.875 | 16 | 16 |
| MKRN1 | 19.25 | 16 | 16 |
| CPSF2 | 14.625 | 16 | 16 |
| PXN | 7.466667 | 15 | 15 |
| LSM3 | 14.4 | 15 | 15 |
| EMG1 | 13.26667 | 15 | 15 |
| CDKN1B | 12.13333 | 15 | 15 |
| COPS8 | 15.4 | 15 | 15 |
| CLTA | 11.86667 | 15 | 15 |
| PPP2CB | 9.4 | 15 | 15 |
| ADRB2 | 11.26667 | 15 | 15 |
| LSM5 | 15 | 14 | 14 |
| ACTR3 | 12.57143 | 14 | 14 |
| CTTN | 11.78571 | 14 | 14 |
| M6PR | 12.92857 | 14 | 14 |
| PGRMC1 | 8.785714 | 14 | 14 |
| ARPC5 | 12.15385 | 13 | 13 |
| NIFK | 12.84615 | 13 | 13 |
| STAM | 11.46154 | 13 | 13 |
| STAM2 | 11.46154 | 13 | 13 |
| GTF2E1 | 11.41667 | 12 | 12 |
| HSPA9 | 3.916667 | 12 | 12 |
| RAP1A | 10.83333 | 12 | 12 |
| CAPZA2 | 10.91667 | 12 | 12 |
| SF3B6 | 17.08333 | 12 | 12 |
| MAPRE1 | 9.5 | 12 | 12 |
| PLRG1 | 17 | 12 | 12 |
| KIF2A | 12.16667 | 12 | 12 |
| LAMTOR2 | 10.16667 | 12 | 12 |
| AJUBA | 15.16667 | 12 | 12 |
| COMMD6 | 14.5 | 12 | 12 |
| PSMB2 | 10.90909 | 11 | 11 |
| MRPS5 | 11.63636 | 11 | 11 |
| C3 | 7.090909 | 11 | 11 |
| MRPL33 | 10.81818 | 11 | 11 |
| ARF4 | 10.63636 | 11 | 11 |
| RAB14 | 7.909091 | 11 | 11 |
| ACTN1 | 7.181818 | 11 | 11 |
| CHERP | 18.18182 | 11 | 11 |
| CCAR1 | 18.18182 | 11 | 11 |
| MYH9 | 11.81818 | 11 | 11 |

**Supplementary Table 3**

**Supplementary Table 3** show key genes in PPI network in purple module.

(Nodes > 10)

| symbol | NeighborhoodConnectivity | NumberOfDirectedEdges | Degree |
| --- | --- | --- | --- |
| RPS11 | 11.45 | 20 | 20 |
| RPS24 | 12.11111 | 18 | 18 |
| RPL26 | 13.11765 | 17 | 17 |
| RPS12 | 12.5 | 16 | 16 |
| MRPL15 | 10.6 | 15 | 15 |
| RPS27L | 13.13333 | 15 | 15 |
| RPL21 | 13.13333 | 15 | 15 |
| RPL18A | 13.53333 | 15 | 15 |
| RPLP2 | 13.13333 | 15 | 15 |
| RPL17 | 13.64286 | 14 | 14 |
| PSMA3 | 6.916667 | 12 | 12 |
| CCT5 | 13.09091 | 11 | 11 |
| EIF3M | 15.27273 | 11 | 11 |

**Supplementary Table 4**

**Supplementary Table 4** show key genes in cyan module. (MM > 0.8, GS > 0.2)

| symbol | moduleColor | GS.HO+ | MMcyan |
| --- | --- | --- | --- |
| ACTR2 | cyan | 0.594959 | 0.957801 |
| ACTR3 | cyan | 0.523754 | 0.894119 |
| ACTR6 | cyan | 0.824133 | 0.896995 |
| AGPAT5 | cyan | 0.351359 | 0.812335 |
| AGPS | cyan | 0.712923 | 0.898263 |
| ALAD | cyan | 0.328277 | 0.812747 |
| APIP | cyan | 0.620954 | 0.91306 |
| ARF4 | cyan | 0.57991 | 0.858893 |
| ATAD1 | cyan | 0.517996 | 0.899881 |
| ATP6V1G1 | cyan | 0.620256 | 0.88885 |
| ATP7B | cyan | 0.636567 | 0.804122 |
| C10orf119 | cyan | 0.603276 | 0.880449 |
| C12orf11 | cyan | 0.766176 | 0.826564 |
| C17orf39 | cyan | 0.756262 | 0.836971 |
| C1orf103 | cyan | 0.506504 | 0.941041 |
| C20orf108 | cyan | 0.433822 | 0.838641 |
| C5orf37 | cyan | 0.420035 | 0.899654 |
| C6orf182 | cyan | 0.254761 | 0.846688 |
| C6orf211 | cyan | 0.28496 | 0.855217 |
| C6orf62 | cyan | 0.681971 | 0.813048 |
| C8orf48 | cyan | 0.513302 | 0.915493 |
| C9orf150 | cyan | 0.508126 | 0.827426 |
| C9orf40 | cyan | 0.28378 | 0.825814 |
| C9orf82 | cyan | 0.36913 | 0.833137 |
| CCDC90A | cyan | 0.689764 | 0.89623 |
| CCNC | cyan | 0.399376 | 0.895215 |
| CDK8 | cyan | 0.248731 | 0.831707 |
| CEP78 | cyan | 0.394214 | 0.905555 |
| CFL2 | cyan | 0.288854 | 0.881097 |
| COQ3 | cyan | 0.622816 | 0.859764 |
| COX7A2L | cyan | 0.576876 | 0.86465 |
| CYP20A1 | cyan | 0.606251 | 0.80787 |
| CYP3A5 | cyan | 0.646344 | 0.803782 |
| CYP51A1 | cyan | 0.752927 | 0.921842 |
| DCK | cyan | 0.724927 | 0.855217 |
| DCUN1D5 | cyan | 0.430732 | 0.861145 |
| DDIT4 | cyan | 0.552989 | 0.810214 |
| DIAPH2 | cyan | 0.586969 | 0.924596 |
| DPH3 | cyan | 0.521223 | 0.838847 |
| DPH3B | cyan | 0.671768 | 0.833569 |
| DSCR3 | cyan | 0.272187 | 0.869196 |
| EDEM1 | cyan | 0.400516 | 0.906922 |
| EID2B | cyan | 0.399463 | 0.802826 |
| ENOPH1 | cyan | 0.509619 | 0.946371 |
| EXOSC3 | cyan | 0.426658 | 0.823803 |
| FAIM | cyan | 0.582462 | 0.825506 |
| FAM124A | cyan | 0.462829 | 0.803645 |
| FAM176A | cyan | 0.419126 | 0.889532 |
| FAM91A1 | cyan | 0.634498 | 0.90306 |
| FERMT2 | cyan | 0.3322 | 0.892695 |
| FMR1 | cyan | 0.359444 | 0.876274 |
| GBP1 | cyan | 0.403568 | 0.863932 |
| GLIPR2 | cyan | 0.373927 | 0.862245 |
| GLMN | cyan | 0.541993 | 0.8207 |
| GPN3 | cyan | 0.49536 | 0.863304 |
| HEATR3 | cyan | 0.425388 | 0.825878 |
| HMGCR | cyan | 0.836909 | 0.878982 |
| IDH3A | cyan | 0.451813 | 0.865212 |
| IER3IP1 | cyan | 0.505302 | 0.830375 |
| IFI16 | cyan | 0.208777 | 0.838703 |
| JRKL | cyan | 0.402673 | 0.81845 |
| KIAA1143 | cyan | 0.334299 | 0.802522 |
| KLHDC10 | cyan | 0.435065 | 0.884018 |
| LGALS3BP | cyan | 0.609417 | 0.850879 |
| LIMD2 | cyan | 0.529891 | 0.861099 |
| LIN7C | cyan | 0.491635 | 0.944603 |
| LPGAT1 | cyan | 0.545963 | 0.805398 |
| M6PR | cyan | 0.366914 | 0.822421 |
| MARCKS | cyan | 0.820017 | 0.877242 |
| MORF4 | cyan | 0.367963 | 0.84103 |
| MORF4L1 | cyan | 0.62127 | 0.937954 |
| MTERFD1 | cyan | 0.674789 | 0.899109 |
| MTMR2 | cyan | 0.662935 | 0.812449 |
| NARS | cyan | 0.347715 | 0.835427 |
| NIPSNAP3B | cyan | 0.726423 | 0.834164 |
| NPTN | cyan | 0.586552 | 0.814254 |
| NR2F2 | cyan | 0.772774 | 0.879027 |
| NUDT15 | cyan | 0.558363 | 0.900958 |
| NXN | cyan | 0.681861 | 0.848317 |
| PALLD | cyan | 0.472237 | 0.895107 |
| PDCL3 | cyan | 0.782721 | 0.880291 |
| PDIK1L | cyan | 0.509025 | 0.843869 |
| PGGT1B | cyan | 0.500885 | 0.928352 |
| PGRMC1 | cyan | 0.525534 | 0.840647 |
| PIP4K2A | cyan | 0.5292 | 0.8868 |
| PLEKHO1 | cyan | 0.379948 | 0.921712 |
| POLR2J2 | cyan | 0.37151 | 0.815587 |
| PPP1CB | cyan | 0.351942 | 0.828584 |
| QPRT | cyan | 0.625646 | 0.806757 |
| RABL3 | cyan | 0.68648 | 0.867557 |
| RBM12B | cyan | 0.529069 | 0.837676 |
| RBM34 | cyan | 0.583461 | 0.833666 |
| RBM7 | cyan | 0.64099 | 0.891173 |
| RIOK1 | cyan | 0.418609 | 0.893699 |
| RNFT1 | cyan | 0.590019 | 0.830061 |
| RPRD1A | cyan | 0.406886 | 0.910349 |
| RPS23 | cyan | 0.597087 | 0.841737 |
| RSRC1 | cyan | 0.37486 | 0.811096 |
| SCD | cyan | 0.795521 | 0.803468 |
| SEL1L3 | cyan | 0.316101 | 0.807011 |
| Septin 11 | cyan | 0.628604 | 0.834851 |
| SERPINH1 | cyan | 0.316328 | 0.852352 |
| SIAH1 | cyan | 0.518302 | 0.86805 |
| SLC25A24 | cyan | 0.653932 | 0.820104 |
| SLC33A1 | cyan | 0.488052 | 0.870011 |
| SLC35A2 | cyan | 0.701739 | 0.8003 |
| SLC35B3 | cyan | 0.30529 | 0.838191 |
| SLC39A9 | cyan | 0.724022 | 0.903037 |
| SLIT2 | cyan | 0.484365 | 0.853791 |
| SNAP23 | cyan | 0.278484 | 0.87694 |
| SPAG16 | cyan | 0.532837 | 0.91709 |
| SUSD3 | cyan | 0.538123 | 0.888525 |
| TADA1 | cyan | 0.324227 | 0.819561 |
| TBC1D9 | cyan | 0.203458 | 0.805496 |
| TIGD2 | cyan | 0.458614 | 0.804008 |
| TMEM123 | cyan | 0.356922 | 0.808167 |
| TMEM30A | cyan | 0.383299 | 0.874326 |
| TPM4 | cyan | 0.498382 | 0.943685 |
| TRDMT1 | cyan | 0.569685 | 0.933543 |
| TRPM7 | cyan | 0.488145 | 0.884505 |
| TSEN15 | cyan | 0.491219 | 0.821107 |
| TUBGCP4 | cyan | 0.525365 | 0.912071 |
| TUSC1 | cyan | 0.558738 | 0.839642 |
| TXNDC9 | cyan | 0.579672 | 0.845256 |
| UBE2NL | cyan | 0.62105 | 0.911719 |
| WDR12 | cyan | 0.350307 | 0.8135 |
| ZCCHC9 | cyan | 0.308616 | 0.84111 |
| ZNF217 | cyan | 0.483125 | 0.869385 |
| ZNF330 | cyan | 0.457392 | 0.860431 |
| ZNF354A | cyan | 0.470409 | 0.832973 |

**Supplementary Table 5**

**Supplementary Table 5** show key genes in purple module. (MM > 0.8, GS > 0.2)

| symbol | moduleColor | GS.HO+ | MMpurple |
| --- | --- | --- | --- |
| ACTB | purple | 0.471453 | 0.83602 |
| ACYP2 | purple | 0.603311 | 0.895092 |
| ALDOA | purple | 0.804816 | 0.882294 |
| ANKRD37 | purple | 0.783305 | 0.846871 |
| ANXA8L2 | purple | 0.567423 | 0.810292 |
| AP1S1 | purple | 0.570176 | 0.802774 |
| ATCAY | purple | 0.849133 | 0.894311 |
| ATP13A3 | purple | 0.395344 | 0.837226 |
| ATP6V0E1 | purple | 0.683435 | 0.909736 |
| ATP7A | purple | 0.556216 | 0.841564 |
| BCL7A | purple | 0.732425 | 0.838351 |
| BOLA3 | purple | 0.669878 | 0.839415 |
| BST1 | purple | 0.549775 | 0.852719 |
| C10orf76 | purple | 0.621383 | 0.804651 |
| C14orf156 | purple | 0.513459 | 0.842908 |
| C1orf25 | purple | 0.777796 | 0.813627 |
| CETN3 | purple | 0.399319 | 0.844949 |
| CNIH | purple | 0.538451 | 0.801976 |
| DCTN6 | purple | 0.474258 | 0.842544 |
| DKK1 | purple | 0.687157 | 0.871822 |
| DNAJC19 | purple | 0.503607 | 0.870835 |
| DYNLRB1 | purple | 0.384553 | 0.841324 |
| ERO1L | purple | 0.410433 | 0.806559 |
| FLJ40504 | purple | 0.65768 | 0.892834 |
| GTF2A2 | purple | 0.493702 | 0.894446 |
| HBXIP | purple | 0.436473 | 0.906724 |
| HSPB8 | purple | 0.518282 | 0.840697 |
| HSPH1 | purple | 0.742578 | 0.823105 |
| KRT18 | purple | 0.680708 | 0.892437 |
| LGR4 | purple | 0.757159 | 0.855347 |
| LOC442249 | purple | 0.650632 | 0.879699 |
| LRRC40 | purple | 0.787379 | 0.819155 |
| LY96 | purple | 0.773144 | 0.875333 |
| MED31 | purple | 0.387353 | 0.827722 |
| MFSD1 | purple | 0.680573 | 0.88268 |
| NAA20 | purple | 0.640495 | 0.864803 |
| NDUFC2 | purple | 0.609478 | 0.868013 |
| NUDT9 | purple | 0.675814 | 0.853813 |
| OSTM1 | purple | 0.782736 | 0.934025 |
| PSMA1 | purple | 0.429554 | 0.847192 |
| PSMA3 | purple | 0.81929 | 0.845045 |
| PTCD3 | purple | 0.472157 | 0.809422 |
| PTS | purple | 0.830975 | 0.863225 |
| ROR1 | purple | 0.545477 | 0.915627 |
| RPS27L | purple | 0.435358 | 0.871322 |
| RWDD1 | purple | 0.507995 | 0.842632 |
| SCARB1 | purple | 0.714184 | 0.815132 |
| SEC11C | purple | 0.694399 | 0.834661 |
| SERF1A | purple | 0.625238 | 0.827926 |
| SH2D5 | purple | 0.557763 | 0.830386 |
| SSBP1 | purple | 0.427048 | 0.817621 |
| TAF9 | purple | 0.728831 | 0.81354 |
| TANC1 | purple | 0.792402 | 0.842512 |
| tcag7.873 | purple | 0.490373 | 0.862793 |
| THAP8 | purple | 0.717014 | 0.838966 |
| TMEM138 | purple | 0.754275 | 0.830742 |
| TMEM167A | purple | 0.526198 | 0.878439 |
| TPP1 | purple | 0.696747 | 0.832338 |
| ZNF259P1 | purple | 0.51334 | 0.83114 |
